# Cervicofacial and mediastinal emphysema following minor dental procedure: a case report and review of the literature

**DOI:** 10.1186/s40463-020-00455-0

**Published:** 2020-08-18

**Authors:** Adnan Busuladzic, Melissa Patry, Laurent Fradet, Valérie Turgeon, Marie Bussieres

**Affiliations:** 1 Université de Sherbrooke, 580 Bowen Sud, Sherbrooke, QC J1G 2E8 Canada; 2 Clinique Dentaire du Carrefour, 2910, boul. Portland, Sherbrooke, QC J1L 1R8 Canada

**Keywords:** Pneumomediastinum, Subcutaneous emphysema, Dental restoration, Necrotizing fasciitis

## Abstract

**Background:**

Subcutaneous cervical emphysema is a clinical sign associated with many conditions, including laryngotracheal trauma, pneumothorax and necrotizing deep tissue infections.

**Case presentation:**

We discuss a case of a 76-year-old man presenting with extensive cervical emphysema a few hours after a minor dental filling procedure. The CT-scan revealed a significant amount of air within the cervical and mediastinal spaces, reaching lobar bronchi. Vitals were within normal values Bloodwork demonstrated an elevation of creatinine kinase (3718; normal < 150) and mild leukocytosis (WBC = 11.6). We decided to proceed to an urgent cervical exploration to exclude necrotizing fasciitis. This revealed air but no tissue necrosis nor abnormal fluid. The patient improved clinically and was discharged two days later with oral antibiotics. Although cervicofacial subcutaneous emphysema following dental procedures has been reported, it is usually less extensive and involving more invasive procedures using air-driven handpieces.

**Conclusion:**

As an otolaryngologist confronted with extensive subcutaneous emphysema following a potential entry route for an aggressive infection, given the seriousness of this diagnosis, the decision of whether or not to perform a diagnostic surgical exploration should remain.

## Introduction

Subcutaneous cervicofacial emphysema is a relatively frequent clinical entity and has a large differential diagnosis including, among others: angioedema and/or anaphylactic reaction, deep neck space infections, necrotizing fasciitis, airway trauma, dental or surgical procedures, pneumothorax or pneumomediastinum. Iatrogenic subcutaneous emphysema can be diagnosed through history and physical examination, combined with the right radiological and laboratory tests, after exclusion of life-threatening pathologies.

The first case of subcutaneous emphysema caused by a dental procedure has been reported in 1900 by Turnbull et al. [1] So far, two reviews have been published in dentistry journals, respectively in 1995 by Heyman et al. [2] and in 2006 by McKenzie et al. [3]. Our objective is to report a severe case of subcutaneous emphysema, to review the last 10 years of literature on the topic and to discuss the management of those patients from an otolaryngologist’s point of view.

## Case report

A 76-year-old male presented to the emergency department in our tertiary care center with left-sided cervicofacial subcutaneous emphysema. The questionnaire revealed he had sustained a routine dental filling of tooth #34 a few hours before. A small retraction cord (#00) was used without an air-driven high-speed hand piece. However, an air syringe was used to do the filling. In that case, a rubber dam could not be placed due to the presence of an old subgingival defective restauration in place. The procedure was done under local anesthesia without any ventilation, positive pressure event or CPAP use. About an hour after, cervical swelling and tenderness progressed. There were no other complaints. He had the same filing with the same procedure on the tooth #44 two weeks before.

He was otherwise known for hypertension, dyslipidemia and moderate chronic renal failure (baseline serum creatinine: 130 μmol/L). He had no history of head and neck pathologies or surgeries. He had known mild allergies to sulfamethoxzaole/Trimethoprime and to amoxicillin, but no to penicillin. The patient was on simvastatin and had no recent change to his medication.

Physical examination revealed extensive, mainly left sided cervicofacial subcutaneous emphysema with associated erythema and tenderness on palpation. Vitals were: blood pressure 195 over 97 mmHg, heart rate 60 bpm and body temperature 37.5 °C.Oral cavity and teeth were unremarkable. There was no evidence of airway obstruction or respiratory distress. The remainder of the physical examination was within normal limits.

**Fig. 1:**
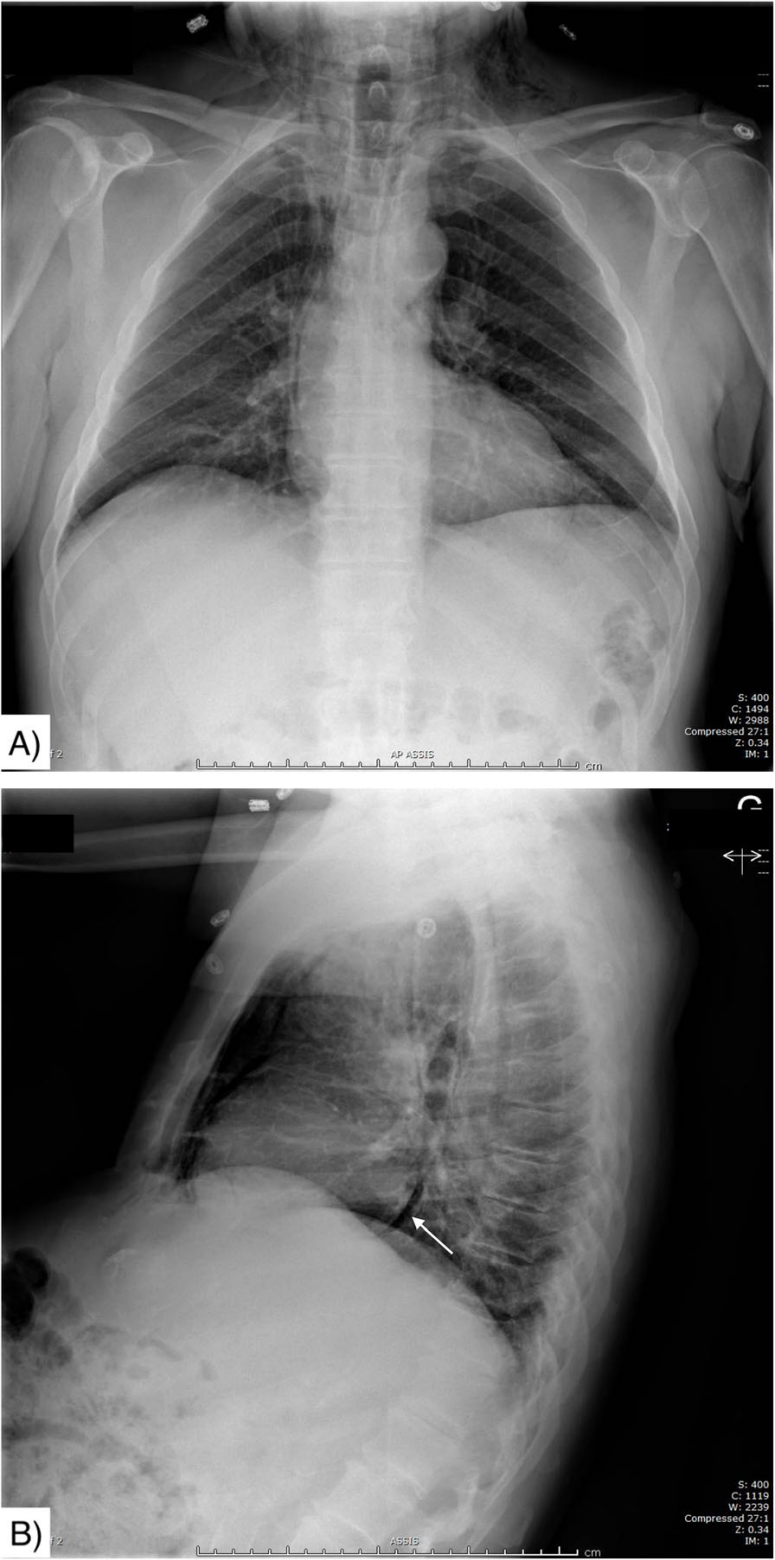
Initial CXR on arrival. **a** Antero-posterior view - important pneumomediastinum **b**) Lateral view - suspected pneumopericardium (white arrow). Both views show diffuse cervical emphysema

Blood tests showed a mild neutrophil-driven leucocytosis (white blood cells count of 11.6 × 10^6^ (normal 3.8–10.6 × 10^6^/mm^3^) with 7.8 × 10^6^ neutrophils) along with a marked elevation of creatinine kinase at 3714 (normal < 185 units/L) and patient’s baseline at 216). C-reactive protein was within normal limits. A chest x-ray (CXR) confirmed diffuse cervical emphysema and pneumomediastinum (Fig. 1).

**Fig. 2:**
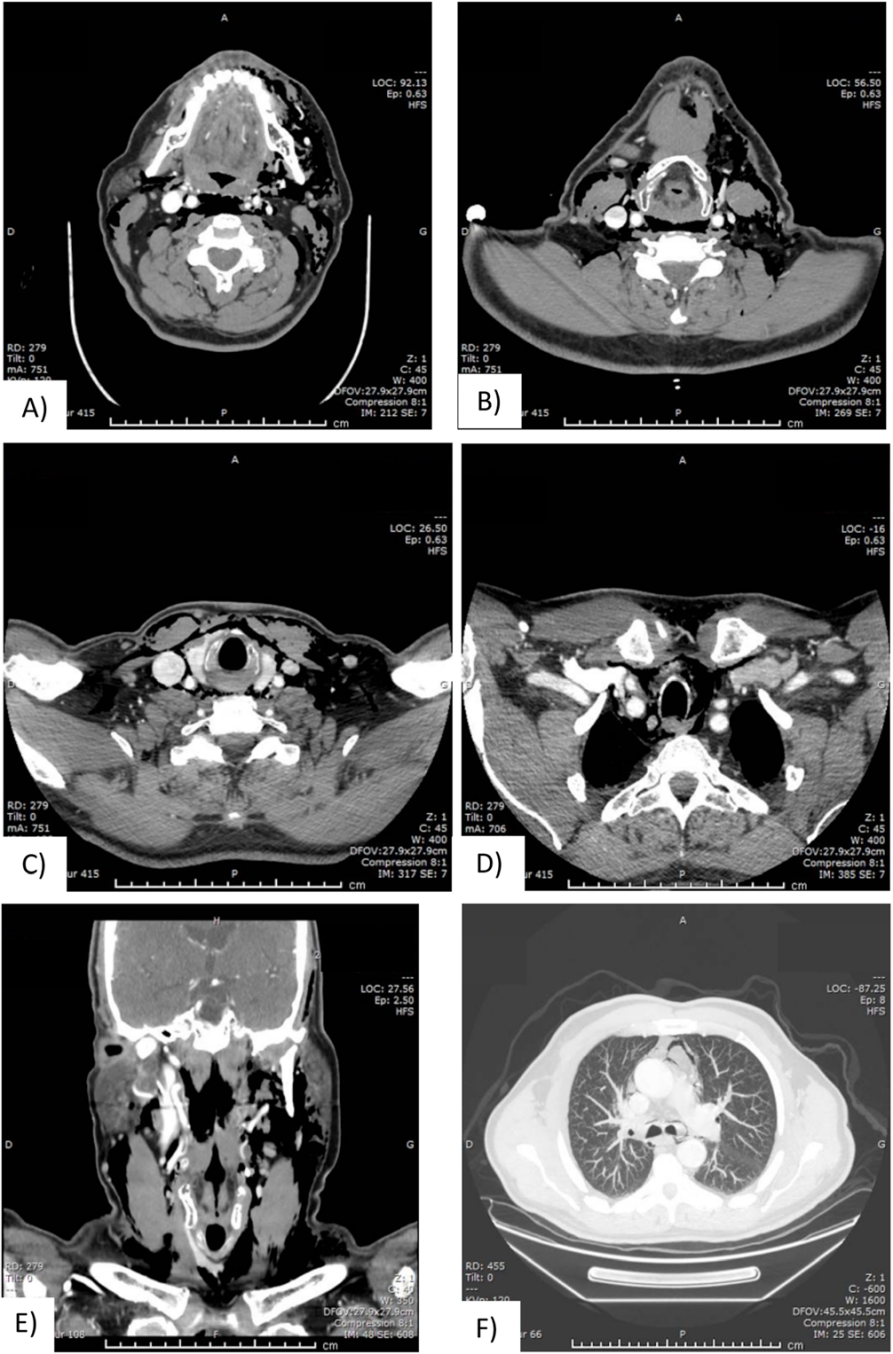
Cervicothoracic CT on arrival. **a** through **f**) Extensive emphysema, involving almost every deep neck and mediastinal spaces

A cervicothoracic computed tomography (CT) was ordered and showed a significant quantity of air in the superficial and deep spaces of the neck and mediastinum reaching the lobar bronchi bilaterally, suspicious of an aggressive infectious process according to the radiologist report (Fig. 2).

**Fig. 3:**
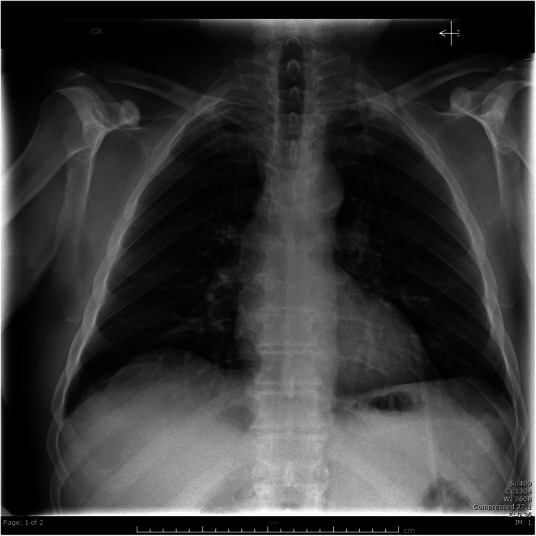
Postoperative day 2 CXR.Postero-anterior view showing significant improvement of subcutaneous emphysem

A developing necrotizing fasciitis could not be ruled out considering the extensive clinical and radiological subcutaneous emphysema associated with the leukocytosis and the significant rise in CK levels. Antibiotic therapy consisting of piperacillin/tazobactam, vancomycin and clindamycin was administered and an urgent surgical cervical exploration was performed, revealing air bubbles that had dissected the involved deep spaces but no evidence of tissue necrosis nor exudative fluid. Hemocultures and surgical wound cultures eventually came back negative. A CXR on postoperative day 2 showed a marked decrease of the cervical subcutaneous emphysema (Fig. 3).

The patient was discharged two days later with moxifloxacin for a total of 7 days. Under infectious disease specialist’s advice, moxifloxacin was chosen because of the patient’s allergy to amoxicillin and of its daily dosage. The patient was seen for follow-up at 3 months and was doing well without any sequelae except the well-healed scar.

## Literature review

A comprehensive review of the English and French literature from 2009 to 2018 was conducted through the PubMed database, using the research terms “dental”, “cervical emphysema” and “dental procedure”, in January 2020. Thirty-eight articles were selected based on their abstract and full text and were analyzed by 2 separate authors (AB and MP). The articles are summarized in Table 1. All patients presented with fascial and/or cervical swelling, and 37 (90.2%) presented within 24 h of the dental procedure. Thirty patients (73.2%) had a procedure involving molar teeth, of which twenty-two (73.3%) were mandibular. Twenty-eight patients (68.3%) had their procedure performed with a dental high-speed handpiece and five (12.2%) with an air-syringe. Thirty-eight patients (92.6%) had thoracic imaging (CXR or CT-scan), of which 27 (65.6%) had intrathoracic air or pneumomediastinum. Thirty-eight (92.6%) also received prophylactic antibiotics. Antibiotic regimen was heterogenous and was not detailed in 25 cases (61%). No complications were noted. No surgeries were performed, and all patients evolved well with resolution of the subcutaneous emphysema.

**Table 1:** Summary of the literature review

Reference	Age (years) / Sex	Procedure / tooth (#)	Suspected cause	Timing of SC emphysema	Imaging modality/ air in mediastinum or intrathoracic	Labs	Hospitalization (#days)/Treatment
Arai & al. (2009) [4]	40/F	Extraction/ 48	HS	1 day	CT / -	N	5 days / Ampicillin
Parkar & al. (2009) [5]	55/F	Endodontic treatment/ left upper molar	HS	1 h	XR/ -	N	1 day / corticosteroids + anti-histaminic + antibiotics
Samuels (2009) [6]	20/F	Extraction/ left lower molar	HS	Immediate	XR/ +	N	admitted / corticosteroids + analgesia + antibiotics
Kim & al. (2010) [7]	40/M	Endodontic treatment / 36	HS	Immediate	CT / +	N	5 days / O2 + antibiotics
Kim & al. (2010) [7]	52/F	Endodontic treatment / 16	HS	Per-procedure	CT /+	N	8 days / antibiotics
Sainsbury & Jaiganesh (2010) [8]	40/M	Endodontic treatment / 27	HS	Per-procedure	XR / -	N	< 1 day (14 h) / O2 + AmoxiClav
Afzali & al. (2010) [9]	16/M	Extraction / 37	HS	1 day	XR + CT / +	WBC 21000	5 days / IV Clinda and ceftazidime
Hsu (2010) [10]	59/F	Endodontic treatment / 38 + 48	HS	1 h	CT /−	N	Antibiotics
Bilecenoglu & al. (2012) [11]	39/F	Extraction / 46	HS	1 day	–	N	N/A / analgesia + antibiotics
Durukan & al. (2012) [12]	45/F	Endodontic treatment / 16	AS + HS	Immediate	XR+ CT/ +	N	3 days/ O2 + metronidazole+ ampicillin
Bergen (2013) [13]	72/F	RDP / molar	HS	Per-procedure	XR / -	N	N/A / Amoxi Clav
Elia & al.(2013) [14]	41/F	Extraction/ 47	HS	Per-procedure	CT/ +	N	7 days/ analgesia + antibiotics
Khandelwal & al. (2013) [15]	4,5/F	Crown preparation / 16	AS + HS	1 h	–	–	- / Amoxicillin
Mitsunaga & al.(2013) [16]	76/F	Laser treatment / 26	Laser	Immediate	CT / +	–	5 days / antibiotics
Olate & al. (2013) [17]	23/F	Extraction / 48	HS	4 h	CT/ -	–	admitted / analgesia + chlorexidine mouth wash + Cefazolin
An & al. (2014) [18]	33/F	Endodontic treatment/ 44	AS	Per-procedure	CT/+	N	5 days / steroids + IV fluids + O2 + clindamycin and switch to ampicillin + metronidazole
Fleischman & al. (2014) [19]	15/F	Extraction / 28	?	Immediate	CT/ -	–	N/A / attempt to decompress the eyelid (30G needle) + antibiotics
Kün-Darbois & al. (2014) [20]	41/F	Extraction / 38	HS	Per-procedure	CT / +	WBC 10370	2 days / -
Paik & al. (2014) [21]	13/M	RDP / 36	HS	Immediate	CT /+	–	1 day / -
Nishimura & al. (2015) [22]	68/M	RDP /?	HS	1 day	XR + CT / +	N	N/A / antibiotics
Picard & al. (2015) [23]	27/M	Extraction / 48	HS	4 days	CT / +	–	4 days / antibiotics
Ocakcioglu & al. (2015) [24]	23/M	Extraction / 48	HS	7 days	CT / +	–	4 days / O2 + antibiotics
Alonso & al.(2017) [25]	73/F	Peri-implant cleaning / 4?	AP	Immediate	CT / -	–	N/A / corticosteroids + antibiotics
Alonso & al.(2017) [25]	43/M	Dental cleaning / 42–43	AP	Immediate	XR / +	–	< 1 day (12 h) / -
Alonso & al.(2017) [25]	62/F	Dental cleaning / 47	AP	Immediate	CT / -	–	N/A / Ibuprofen + antibiotics
Lee & al. (2017) [26]	59/F	RDP / 44	HS	Immediate	XR + CT/ +	N	8 days / O2 + ampicillin + TMP SMX
Ramnarine & Dubin (2017) [27]	28/F	RDP / 14 + 20 + 21	HS	Immediate	XR + CT/+	N	< 1 day (12 h) / antibiotics
Tan & Nikolarakos (2017) [28]	33/F	Extraction / 46	HS	1 day	XR / -	WBC 10000	2 days / analgesia + antibiotics
Thompson & Gohil (2017) [29]	50/M	Extraction / 38	?	4–6 h	XR / +	N	Admitted/ saline nebulisers + antibiotics
Chien (2018) [30]	59/F	RDP / 44 + 46	HS	Immediate	–	–	N/A / antibiotics
Jeong & al. (2018) [31]	60/F	Crown preparation / 15	HS	1 h	XR + CT/ +	N	4 days / O2 + antibiotics
Lee & al.(2018) [32]	51/F	Peri-implant cleaning / 12	AS	Per-procedure	XR + CT/ +	N	13 days / O2 + analgesia + antibiotics
Liu & Lin (2018) [33]	22/M	Extraction / 38	?	1 week	XR + CT/ +	Elevated CRP + WBC	Admitted /Amoxi-Clav
Tay & Loh(2018) [34]	18/M	Extraction / 18 + 28 + 38 + 48	HS	1 day	XR + CT/ +	–	5 days/ O2 + antibiotics
Tenore & al. (2017) [35]	60/F	Endodontic treatment / 22	AS	Per-procedure	CT / -	–	Admitted / corticosteroid + analgesia + antibiotics
Cuccia & al [36].	30/F	Extraction / 37	HS	Immediate	CT / +	N	7 days / corticosteroids + tazocin/cubicin + bed rest
Fehrle & al [37].	32/M	Extraction / 48	?	Weeks	CT / +	CRP 75	Admitted / antibiotics
Mascarenhas & al [38].	43/M	RDP / 47	HS	Immediate	XR / -	–	N/A / Amoxicilin
Paschos & al [39].	17/F	Extraction / 38	HS	30 min	CT / +	N	3 days / antibiotics
Rad et & [40].	36/M	Extraction / 37	HS	Immediate	XR / +	N	N/A / antibiotics
Rawlinson & al [41].	40/F	RDP / upper and lower molar	?	1 day	CT / +	WBC 12500	1 day / antibiotics

*F* Female, *M* Male, *RDP* restorative dental procedure, *HS* High speed handpiece, *AP* Air polishing, *AS* air syringe, *XR* X-Ray, *CT* computed tomography scan, *WBC* white blood cell count, *CRP* C-Reactive protein, *HBP* High blood pressure, *SC* subcutaneous, *N* normal

## Discussion

The association between dental procedures and cervicofacial emphysema has been described in the dental literature. Even though every tooth may be implicated, mandibular molars are more frequently involved, for they have a closer relationship with head and neck deep spaces. The buccal, sublingual and submandibular spaces are intimately connected with the roots of these molars. The supra-hyoid spaces are contiguous with infra-hyoid spaces, notably the parapharyngeal and retropharyngeal spaces, which can lead to the mediastinal compartment. Different procedures have been associated with cervicofacial emphysema, ranging from endodental treatment to teeth extractions, even hygiene procedures [42]. Use of air syringes, or more frequently air-driven dental handpieces, which inject air at high pressure, are prominent risk factors [3]. With their use, air can dissect in the soft tissues exposed around the tooth and spread through the deep neck spaces. Dentists can use special equipment, like rubber dams, to isolate the tooth in order to prevent such complications.

Given the eventuality of a dental iatrogenic cause being most probable based on history, surveillance in the emergency department or admission can be considered based on the clinician’s judgment. However, invasive infections, such as necrotizing fasciitis or mediastinitis, should be considered as they are infrequent but potentially catastrophic if not diagnosed promptly. Both are also known complications of the dental procedure itself [26]. In our review, most but not all patients received prophylactic antibiotics. The choice of the antibiotic, the route of administration and the duration of the treatment were heterogenous. Because of its adequate coverage of the buccal flora, penicillin is an adequate first choice, and it is what was chosen in most of the reported cases [1]. We found no case of significant infection, as was the case for McKenzie et al. [3]

Among other treatment modalities that have been reported, steroids have been used empirically in 5 patients to decrease edema and inflammation. Antihistamines were used in only 1 patient to treat empirically for a local anesthetic allergic reaction. However, depending on the clinical context, if an anaphylactic reaction or angio-edema is suspected, epinephrine, steroids and antihistamines should be administered in a timely fashion [3] Oxygen supplementation was administered in 7 patients. Although no study was done to evaluate the efficacy of 100% O2 supplementation in case of subcutaneous emphysema, its use is extrapolated from pneumothorax cases: using 100% O2 accelerates the resorption of pneumothorax by reducing nitrogen gas pressure in pleural capillaries thus promoting resorption of air (mostly nitrogen) from the pleural space [8, 43, 44]. Despite the patient’s well-being and normal CRP, the choice of performing surgical exploration was not instinctive but made mainly because of the significant CK elevation and the radiologist’s report raising a high suspicion index of necrotizing fasciitis. The dental procedure could have been the entry route for an aggressive infection even if, in retrospect, this was not the case.

## Conclusion

The use of high-speed dental handpieces and air-syringes during dental procedures can infrequently precipitate extensive subcutaneous emphysema. Clinical history and paraclinical investigation are keys to making the right diagnosis. In cases of iatrogenic subcutaneous emphysema related to dental procedure, conservative treatment has shown to be a safe option. Nevertheless, high clinical suspicion is warranted for an invasive necrotizing infection, given the seriousness of this eventuality but the choice between close observation or surgical exploration should rely on the clinician’s judgement.

## Data Availability

All data generated or analyzed during this study are included in this published article.

## Abbreviations

AP: Air-polishing
CT: Computed tomography
CXR: Chest x-ray
F: Female
HBP: High blood pressure
HS: High-speed handpiece
M: Male
RDP: Restorative dental procedure
WBC: White blood cell
